# Mechanism of biogenic amine synthesis of *Enterococcus faecium* isolated from Sanchun ham

**DOI:** 10.1002/fsn3.2820

**Published:** 2022-03-14

**Authors:** Yunhe Zhang, Bo Shan, Jiashun Gong, Yongjin Hu

**Affiliations:** ^1^ 12616 Collage of Food Science and Technology Yunnan Agricultural University Kunming China

**Keywords:** alanyl‐leucine, *Enterococcus faecium*, quorum sensing, Sanchuan ham, tyramine

## Abstract

Sanchuan ham, produced in Yunnan, China, is food with ethnic characteristics favored by consumers. However, it can contain biogenic amines such as tyramine that are harmful to health, and the synthesis mechanism of biogenic amines in Sanchuan ham is not clear. This study focuses on the regulation of biogenic amine synthesis by quorum sensing. We used high‐performance liquid chromatography to detect the content of biogenic amine in different kinds of ham and found that the content of biogenic amine in Sanchuan ham was higher than that in others. Tyramine‐producing strain isolated from Sanchuan ham was identified as *Enterococcus faecium*. By monitoring the growth and tyramine synthesis of *Enterococcus faecium* under cultured conditions, the results found that high temperature and low salt increased tyramine production by *E*. *faecium*. After seven exogenous amino acids were applied to *E. faecium*, only tyrosine could promote the production of tyramine in *E. faecium*, and tyramine could not be synthesized in *E. faecium* until a certain amount was reached, indicating the presence of microbial quorum sensing signal molecules in the synthesis of tyramine in *E. faecium*. Untargeted metabolomics analysis of the differential metabolites produced by *E. faecium* showed that the contents of some peptides, especially alanyl‐leucine, were significantly increased. Further experiments with synthetic alanyl‐leucine illustrated that alanyl‐leucine activated the expression of tyrosine decarboxylase (tyrDC), thereby regulating the synthesis of tyramine by *E*. *faecium*. Alanyl‐leucine acted as quorum sensing signal molecules for biogenic amine synthesis by *E*. *faecium*, which provided a theoretical basis for reducing biogenic amine accumulation in ham. It is beneficial to control the content of biogenic amines in ham in the future.

## Graphical abstract

Alanyl‐leucine could be the quorum sensing signal molecule of *Enterococcus faecium* associated with tyramine production, which will be beneficial to control tyramine production of *E. faecium* by inhibiting quorum sensing in the future, thereby inhibiting the content of tyramine in ham, and providing a new idea for controlling the quality of ham.

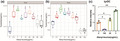

## 1 INTRODUCTION

In China, the process of dry curing ham is prevalent in several regions, including Yunnan Province (Lin et al., 2020). Yunnan is a well‐known production area of various types of fermented foods including dry‐cured ham such as Sanchuan ham (Liu et al., 2019). As dry‐cured ham is generally categorized by its origin, Sanchuan ham is produced in Sanchuan dam, Lijiang City, Yunnan Province, China. Sanchuan ham has a long history and is a traditional food of local minorities such as Naxi and Lisu nationality, which is widely loved by consumers. It is made by drying fresh pig hind legs for 1 day and covering them with a layer of salt, followed by spraying of corn wine and repeated rubbing. Then, the ham was salt cured for the second time for 20 days and wrapped with wet white cotton paper. Subsequently, the ham was hung and air‐dried for 2 months. The main difference between the production processes of Sanchuan ham and other hams is that the dried ham is placed in a big bamboo basket with better air permeability, which is then tightly clamped with plant ash (Hu et al., 2018). The plant ash is alkaline, which can absorb the moisture remaining in the ham and prevent spoilage by insects. As a king of traditional fermented meat products, ham has rich proteins and microorganisms, which induce the production of biogenic amines and endangers the health of consumers (Ashaolu et al., 2021; Suzzi & Gardini, 2003). During salting of Sanchuan ham, natural fermentation is performed under environmental conditions (Wang et al., 2021). Microorganisms in the fermentation process mostly come from the bacteria in the environment and the dominant bacteria existing in the ham (Martínez‐Onandi et al., 2019). Some microorganisms have amino acid decarboxylase activity, such as lactic acid bacteria, which can decarboxylate with the amino acids in the ham to form biogenic amines (Ozogul & Hamed, 2017). The formation mechanism of biogenic amines in Sanchuan ham is still unclear and needs to be studied.

Biogenic amines are organic nitrogen‐containing compounds of low molecular weight that are difficult to volatilize (Brink et al., 1990; Halasz et al., 1994; Vasconcelos et al., 2021). The ingestion of a high concentration of biogenic amines is harmful to human health and may result in poisoning in severe cases (Perin & Nero, 2017; Santos, 1996). Biogenic amines possess vascular activity and play important roles in vivo (Alvatez & Moreno‐Arribas, 2014; Jaguey‐Hernández et al., 2021). However, the risk of biogenic amine poisoning increases when large amounts of biogenic amines are ingested or their natural mechanisms of catabolism are inhibited; for example, when amine oxidase activity is inhibited in people with genetic defects, or in those who consume alcohol or antidepressants (Biji et al., 2016; Zaman et al., 2011). Therefore, the mechanism via which biogenic amines are produced should be studied to inhibit their production in food products.

Microorganisms secrete low molecular weight substances in the environment. With an increase in the number of microorganisms, the amounts of these secreted substances reach the threshold, which is recognized by the corresponding receptors on the microorganisms, triggering or inhibiting the expression of specific genes (Mukherjee & Bassler, 2019). This results in the synchronization of the behaviors of all members of the population, which adjust to the changes in the environment as a feedback response (Kareb & Ader, 2020). By sensing the concentration of signal molecules, microorganisms regulate the signal transduction pathways regulating gene expression and physiological functions of microorganisms, which is called quorum sensing (Miller & Bassler, 2001). Bacteria can communicate with each other, coordinate the activities within bacterial communities, and regulate the relationship among different bacteria via signal molecules (Fan et al., 2022). The production of biogenic amines in microorganisms is also regulated by the quorum sensing system. Diketopiperazines (DKPs), a class of quorum sensing molecules, promote the formation of biofilms, expression of genes encoding trimethylamine oxide reductase and ornithine decarboxylase, and production of trimethylamine, putrescine, and extracellular proteases in *Shewanella* and *Shewanella aquimarina* (Fu et al., 2018; Zhu et al., 2015, 2016). *Enterococcus faecium* is a tyramine‐producing microorganism isolated from Sanchuan ham (Zezong, 2018). However, how the microbial quorum sensing system regulates tyramine production by *Enterococcus* *faecium* in ham is not understood.

In this study, the conditions of producing biogenic amines from *E. faecium* isolated from Sanchuan ham and the molecular mechanism of biogenic amines synthesis based on microbial quorum sensing were studied. Our observations will lay a theoretical basis for controlling amine production in Sanchuan ham and provide a scientific basis for developing healthy and safe ham products.

## 2 MATERIALS AND METHODS

Nine different traditional ham samples Spanish ham (Zaragoza, Spain), Italian ham (Parma, Italy), Rugao ham (Rugao, Jiangsu, China), Xuanwei ham (Xuanwei, Yunnan, China), Nuodeng ham (Nuodeng, Yunnan, China), Dahe Blake Pig ham (Qujing, Yunnan, China), Heqing ham (Heqing, Yunnan, China), Jinhua ham (Jinhua, Zhejiang, China), and Sanchuan ham (Lijiang, Yunnan, China) were purchased from Kunming supermarkets (Wal‐Mart, Carrefour and Metro); alanyl‐leucine, L‐glycyl‐L‐hydroxyproline, Hydroxyprolyl‐methionine, and Prolyl‐methionine were synthesis by Hangzhou ONTORES biotechnology company; L,L‐cyclo(leucylprolyl; Solarbio, China); tyrosine, tryptophan, histidine, arginine, phenylalanine, lysine, and ornithine (Solarbio, China).

### 2.1 Methods

#### 2.1.1 Detection of biogenic amines in hams

The biogenic amine contents of ham were determined by using high‐performance liquid chromatography (HPLC; Thermo Fisher Scientific) according to Moret and Zhang (Huichao et al., 2019; Moret & Conte, 1996). One milliliter of each bacterial culture broth was uniformly mixed with 0.1 M HCl. This mixture was centrifuged at 6480 *g* for 10 min (4°C) and the supernatant was filtered. The filtrate extract (1 ml) was placed in a 5‐ml volumetric flask. Then, sodium hydroxide (2 N, 200 μl), saturated sodium bicarbonate (300 μl), and dansyl chloride solution (10 mg/ml) were added to the sample extract. After incubation at 60 for 15 min in the dark, 100 μl of ammonia was added to the reaction mixture for the removal of residual dansyl chloride. After 30 min at ambient temperature, the volume of the reaction mixture was adjusted to 5 ml with acetonitrile. This reaction mixture was centrifuged for 5 min at 405 *g*. The supernatant was filtered with a 0.22 μm syringe filter with a PVDF membrane for HPLC analysis. The separation was carried out on a C18 column (Spherisob 2.5 μm ODS, 250 cm 4.6 mm internal diameter) and the peaks were detected at 254 nm with a diode array detector. A gradient elution program was used with a mixture of acetonitrile as solvent A and water as solvent B. The gradient elution procedure was 35%A + 65%B for 1 min, 20%A + 80%B for 5 min, 10%A + 90%B at 6 min, and 8%A + 92%B for 16 min. The standard amine samples HIS, TRY, TYR, PUT, PHE, CAD, SPD, and SPE were purchased from Sigma.

#### 2.1.2 Isolation and identification of *E. faecium* from Sanchuan ham

The screening method is according to the work of (Lu et al., 2015). Using aseptic techniques, 20 g Sanchuan ham were homogenized in 180 ml of sterile normal saline. After shaking at 200 r/min for 1 h in a stomacher, 1 ml of the suspension was inoculated into the enrichment broth (MRS; LuQiao Co) for 24 h at 37°C. The enrichment culture was serially diluted in triplicate (1:10) in peptone saline, and 1 ml of each dilution was inoculated onto the lower screen medium. After cultivation at 37°C for 72 h, the upper color development medium (50°C) was layered onto the lower detection medium.

Genomic DNA of amino acid decarboxylase‐positive bacteria was extracted using GenElute^™^ kit (Tiangen Biotech Co., Ltd) according to the manufacturer's instructions, and then suspended in 100 μl of TE buffer and stored at −20°C. The primers 27F (AGTTTGATCMTGGCTCAG) and 1492R (GGTTACCTTGTTACGACTT) were used to amplify the V6‐V8 regions of the bacterial 16S rDNA. GoTaq Green Master Mix (Promega) was used in the PCR reaction. The amplification reactions were carried out in a 25 μl reaction volume containing 12.5 μl GoTaq Green Master Mix, 0.4 μl of each primer (10 pmol/ml), 1 μl DNA template, and 9.5 μl dd H_2_O. The samples were amplified in a BioSci PCR system at 98°C for 2 min, 35 cycles of 98°C for 10 s, 55°C for 15 s, and 72°C for 15 s, followed by a final step for 5 min. The sequences recovered were aligned to 16S rDNA fragments available from the National Center for Biotechnology Information databases (NCBI), searches in BLAST from GenBank were used to find the closest known relatives of the partial 16S rDNA sequences.

#### 2.1.3 Activation of strains

*Enterococcus faecium* stored in glycerol at −80°C was cultured on mannitol salt agar (MRS) at 30°C for 24 h, while another ring was inoculated in MRS broth at 30°C. After culturing for 24 h, 1% of the broth culture was inoculated in fresh MRS broth. After incubating for 24 h, the culture was used for subsequent experiments. All culture media were obtained from Lu Qiao Company.

#### 2.1.4 Effect of fermentation conditions on the production of biogenic amines by *E. faecium*

The strain was cultured for 24 h, and the absorbance and biogenic amine content were determined every 2 h. Temperature, NaCl content, pH of culture medium, the absorbance, and biogenic amine content in the bacterial supernatant in each group were determined after 24 h. The absorbance at 600 nm was determined using a microplate reader (Thermo Fisher Scientific).

#### 2.1.5 Effects of exogenous amino acids on the production of biogenic amines by *E. faecium*

Nine experimental groups were set up. Seven amino acids, namely tryptophan, histidine, arginine, phenylalanine, lysine, ornithine, and tyrosine, were added to seven groups. The eight group contained all seven amino acids, while amino acids were not added to the last group, which was used as the control group. The biogenic amine content in bacterial liquid was detected after 24 h of culture.

Culture media containing different concentrations of tyrosine were prepared, inoculated with *E. faecium*, and cultured for 12 h. The biogenic amine content in bacterial liquid was detected.

#### 2.1.6 Verifying the regulation of the production of biogenic amines by quorum sensing signal molecules of *E. faecium*

*Enterococcus faecium* cultured in liquid medium for 1, 3, 6, 9 and 24 h were used as the liquid medium (LM) groups. The supernatants of the LM groups were filtered using 0.22 μm filter membrane, inoculated with 1% bacteria, and then cultured for 1 h, which were then considered the supernatant (S) groups. The content of biogenic amine in the supernatant of the LM and S groups was detected. Tyrosine decarboxylase (*tyrDC*) and tyrosine transporter (*tyrP*) expression was determined using reverse transcription polymerase chain reaction (qRT‐PCR). Two milliliters of the testing sample were centrifuged at 8099 *g* at 4°C for 10 min to precipitate the bacteria, and the total RNA of bacteria was extracted using the Trizol method. The concentration of total RNA and the ratio of its absorbance at 260 and 280 nm was determined using a micro nucleic acid quantitative instrument. After the concentration and purity of RNA met the requirements, cDNA was synthesized using a cDNA synthesis kit for removing the remaining genomic DNA. Ten microliters of 2× SuperReal PreMix Plus was added to the enzyme‐free octal tube. Then, 0.6 µl forward primer, 0.6 µl reverse primer, 2 µl cDNA template, and enzyme‐free water was added to make up the volume to 20 µl. After brief centrifugation for mixing the reaction components, PCR was performed using the following amplification cycle: predenaturation at 95°C for 15 min; denaturation at 95°C for 10 s, 40 cycles; and annealing/extension at 60°C for 32 s. Three parallel samples were analyzed in each group.

#### 2.1.7 Nontarget metabolomics detection using ultrahigh‐performance liquid chromatography

Metabolite in the supernatant with *E. faecium*, which was culture for 1, 3, and 9 h, was detected by ultrahigh‐performance liquid chromatography. Metabolite Extraction: Fifty microliters sample was transferred to an Eppendorf tube. After adding 200 μl of extract solution (acetonitrile: methanol = 1:1, containing isotopically labeled internal standard mixture), the samples were vortexed for 30 s, sonicated for 10 min with an ice‐water bath, and incubated for 1 h at −40°C to precipitate the proteins. Then, the sample was centrifuged at 8099 *g* for 15 min at 4°C. The resulting supernatant was transferred to a fresh glass vial for analysis. The quality control (QC) sample was prepared by mixing equal aliquots of the supernatants from all samples. LC‐MS/MS analysis: Liquid chromatography–tandem mass spectrometry (LC‐MS/MS) analyses were performed using a UHPLC system (Vanquish, Thermo Fisher Scientific) with a UPLC BEH Amide column (2.1 mm × 100 mm, 1.7 μm) coupled to Q Exactive HFX mass spectrometer (Orbitrap MS, Thermo). The mobile phase consisted of 25 mmol/L ammonium acetate and 25 ammonia hydroxide in water (pH = 9.75) for phase A and acetonitrile for phase B. The analysis was performed using an elution gradient as follows: 0 ~ 0.5 min, 95% B; 0.5 ~ 7.0 min, 95% ~ 65% B; 7.0 ~ 8.0 min, 65% ~ 40% B; 8.0 ~ 9.0 min, 40% B; 9.0 ~ 9.1 min, 40% ~ 95% B; and 9.1 ~ 12.0 min, 95% B. The column temperature was 30°C. The autosampler temperature was 4°C and the injection volume was 3 μl. The QE HFX mass spectrometer was used for its ability to acquire MS/MS spectra in the information‐dependent acquisition (IDA) mode controlled by the acquisition software (Xcalibur, Thermo). In this mode, the acquisition software continuously evaluates the full‐scan MS spectrum. The ESI source conditions were set as follows: sheath gas flow rate, 50 Arb; Aux gas flow rate, 10 Arb; capillary temperature, 320°C; full MS resolution, 60,000; MS/MS resolution, 7500; collision energy, 10/30/60 in NCE mode; and spray voltage, 3.5 kV (positive) or −3.2 kV (negative).

#### 2.1.8 Effect of alanyl‐leucine on tyramine production by *E. faecium* and *tyrDC* expression

Different concentrations of alanyl‐leucine solutions were prepared and added to *E. faecium* culture solution for 1 and 3 h. The supernatant was taken after centrifugation to determine the content of tyramine. After 3 h of culture, the bacteria were centrifuged and *tyrDC* expression was determined according to 2.2.6.

### 2.2 Data analysis

Data from the experiments are expressed as means ± SEM. Data were analyzed using one‐way analysis of variance (ANOVA) or Student's *t*‐test of SPSS22.0 (IBM) and Prism8 (GraphPad Software). *p* < .05 was considered significant. Nontargeted metabolomics data were plotted using SIMCA14.

## 3 RESULTS AND ANALYSIS

### 3.1 Contents of biogenic amine in hams purchased in Kunming supermarkets

Eight kinds of biogenic amines were detected in nine kinds of hams, which were purchased from Kunming supermarkets, and the results were shown in Table 1. Tyramine was detected in Jinhua ham, Heqing ham, and Sanchuan ham, and the content of tyramine in Jinhua ham was the highest (6.87 ± 0.14 mg/100 g). Putrescine was detected in Sanchuan ham, Hengfa Xuanwei ham, Jinhua ham, and Rugao ham, and the content of putrescine in Jinhua ham was the highest (8.27 ± 1.01 mg/100 g). Histamine was detected in Heqing ham, Hengfa Xuan Wei ham, Parmar ham, Nuodeng ham, and Sanchuan ham. Except for Dahe black ham, Heqing ham, and Spanish ham, the cadaverine was detected in all the other hams. The content of total biogenic amine in Sanchuan ham was 30.21 ± 4.82 mg/100 g, which was the highest among the nine samples tested in this study.

**TABLE 1:** Results of determination of eight kinds of biogenic amines in hams

	Putrescine (mg/100 g)	Cadaverine (mg/100 g)	Histamine (mg/100 g)	Tyramine (mg/100 g)	Spermidine (mg/100 g)	Spermine (mg/100 g)	Total biogenic amine (mg/100 g)
Sanchun Ham	4.57 ± 0.43	1.09 ± 0.02	0.87 ± 0.11	0.97 ± 0.11		18.22 ± 3.67	30.21 ± 4.82
Dahe Blake Pig Ham						19.14 ± 8.65	19.14 ± 8.65
Nuodeng Ham		0.71 ± 0.07	0.99 ± 0.02		1.98 ± 0.97	15.7 ± 1.38	19.46 ± 1.75
Hengfa Xuanwei Ham	3.79 ± 0.49	1.24 ± 0.12	2.60 ± 1.59			12.10 ± 2.37	18.46 ± 4.20
Heqing Ham			4.16 ± 1.00	1.08 ± 0.26	0.89 ± 0.45	15.82 ± 1.88	21.9 ± 2.07
Jinhua Ham	8.27 ± 1.01	1.13 ± 0.09		6.87 ± 0.14		13.11 ± 1.25	29.38 ± 0.63
Rugao Ham	5.98 ± 1.478	1.18 ± 0.09			1.16 ± 0.30	15.68 ± 1.55	23.88 ± 3.77
Italian Ham		0.73 ± 0.03	1.19 ± 0.11		0.69 ± 0.56	12.69 ± 0.62	15.3 ± 1.19
Spanish Ham					0.51 ± 0.30	13.53 ± 2.26	14.04 ± 2.50

### 3.2 Screening and identification of bacteria producing biogenic amines from Sanchuan ham

**FIGURE 1:**
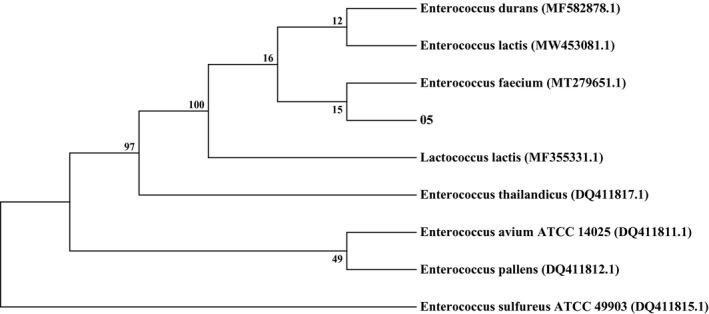
Phylogenetic tree for species identification. O5 was the bacteria separation of Sanchuan ham which produced tyramine

Microorganism is the key factor of ham fermentation; to explore the cause of high biogenic amines content in Sanchuan ham, a strain with high tyramine production was obtained from ham utilizing isolation and identification. The strain‐producing biogenic amines in Sanchuan ham were screened by double culture medium chromogenic method and a strain‐producing tyramine was found. The strain was purple in the Gram staining test and identified as Gram‐positive bacteria. The strain was identified as *Enterococcus faecium* by 16SrDNA sequencing, and the similarity with the NCBI database was 100%, the accession number is MT279651.1. Figure 1 was a phylogenetic tree for species identification.

### 3.3 Effect of fermentation conditions on the biogenic amines production by *E. faecium*

**FIGURE 2:**
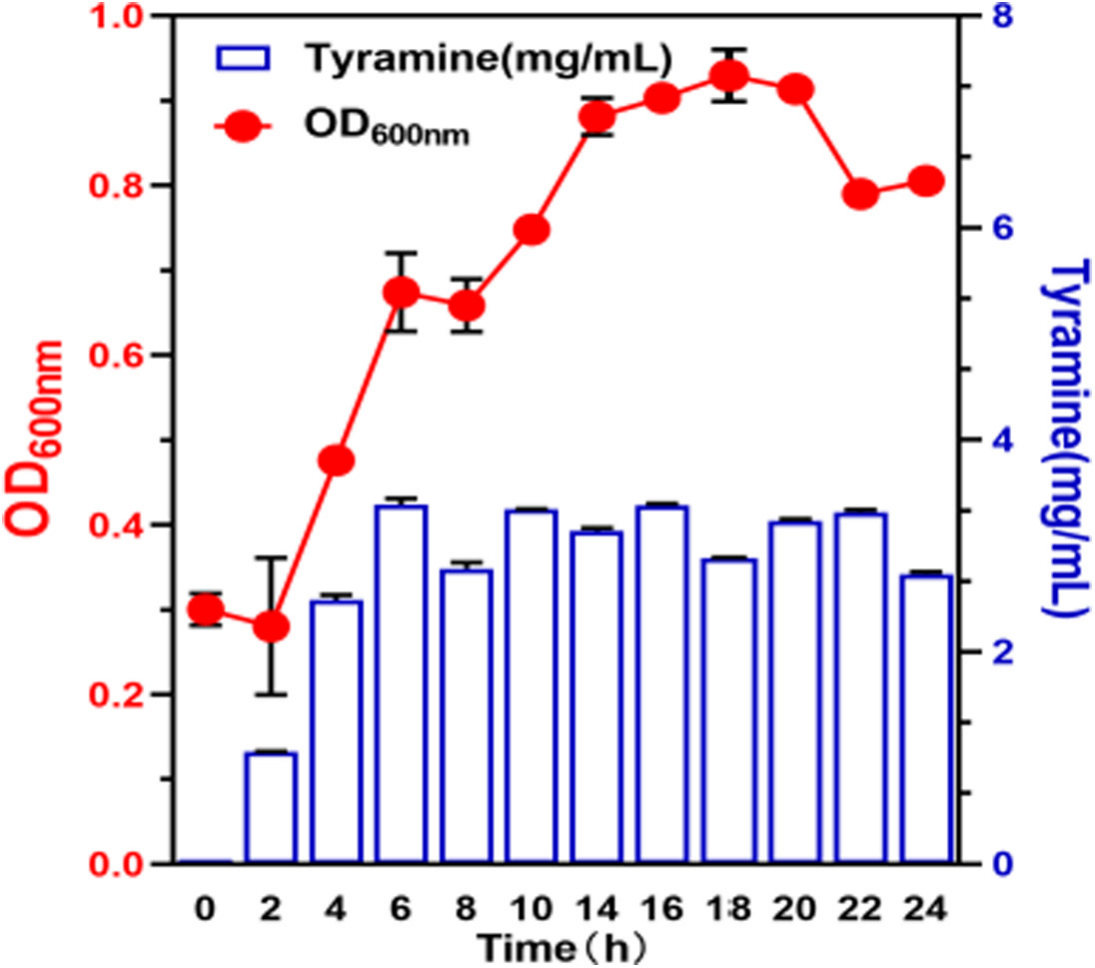
Effect of cultural time on the *Enterococcus faecium* of growth and tyramine synthesis

**FIGURE 3:**
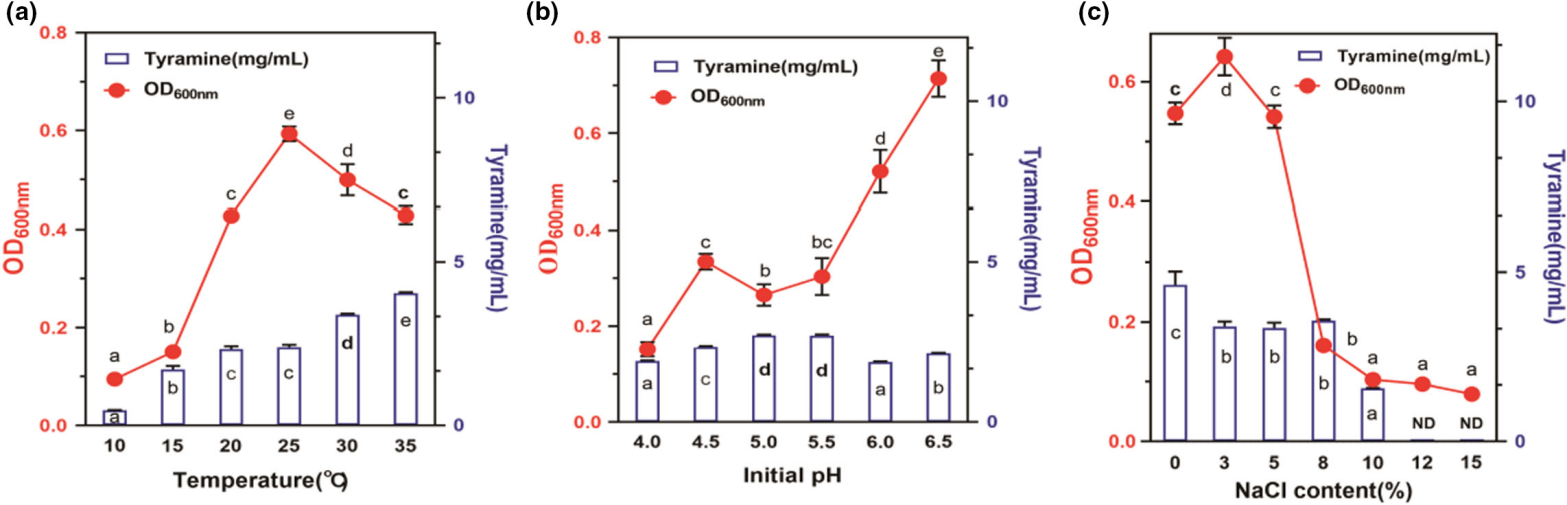
Effect of different cultural conditions on the *Enterococcus faecium* of growth and tyramine synthesis. Different letters in the figures indicate different groups with significant differences (*p* < .05). “ND” means “No Detection”

Figure 2 shows that after 4 h of culture, the density of *E. faecium* started to increase considerably, together with an increase in the OD value and a sharp increase in tyramine content. The tyramine content tended to stabilize after 10 h. Increase in culture temperature was beneficial for the growth of *E. faecium*, and 25°C was optimum for growth. However, a large amount of tyramine was synthesized at 30–35°C (Figure 3a). pH 6.5 was optimum for the growth of *E. faecium*, and the tyramine content was highest at pH 5.0–5.5 (Figure 3b). These results indicated that different initial pH values significantly affected the tyramine production capacity of *E. faecium*. NaCl concentration considerably affected *E. faecium* growth and tyramine content. Growth of *E. faecium* was inhibited significantly when NaCl concentration was higher than 5%; tyramine formation was inhibited when the amount of NaCl was higher than 8% (Figure 3c). These results suggested that increasing the amount of NaCl (>8%) during ham curing may inhibit the growth of *E. faecium* and tyramine synthesis.

### 3.4 Effect of exogenous amino acids on biogenic amines production by *E. faecium*

**FIGURE 4:**
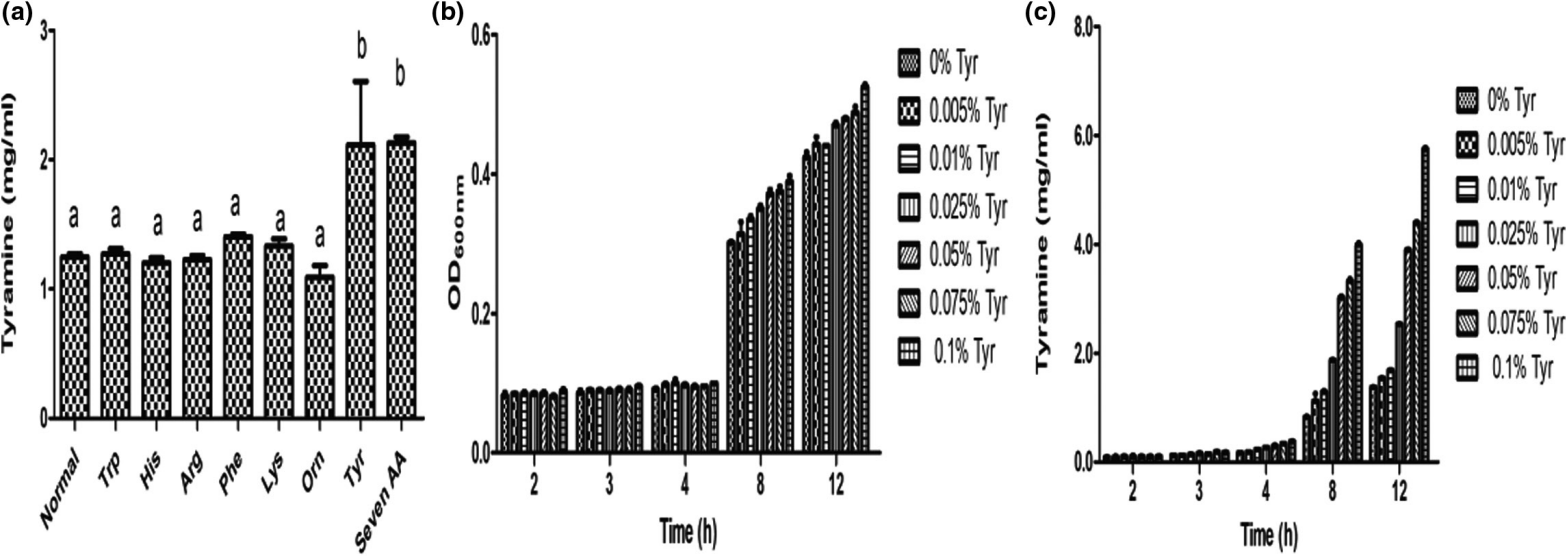
(a) Exogenous amino acid species effect of tyramine synthesis of *Enterococcus faecium*. Tyrosine content effect of growth (b) and tyramine synthesis(c) of *Enterococcus faecium*. Different letters in the figures indicate different groups with significant differences (*p* < .05)

As shown in Figure 4a, the addition of exogenous tryptophan, histidine, arginine, phenylalanine, lysine, and ornithine did not significantly affect the synthesis of tyramine by *E. faecium* (*p* > .05). However, tyrosine addition stimulated the production of a large amount of tyramine. When the culture time was within 4 h, tyrosine addition negligibly affected tyramine synthesis. However, tyramine synthesis increased considerably when the culture lasted for more than 8 h (characterized by high proliferation of *E. faecium*), which increased with tyrosine concentration (Figure 4b,c). Tyrosine stimulates *E. faecium* to synthesize more tyramine. These results suggest that tyrosine is a precursor or substrate for tyramine synthesis.

### 3.5 Verification of regulation of the production of biogenic amines by quorum sensing signal molecules of *E. faecium*

**FIGURE 5:**
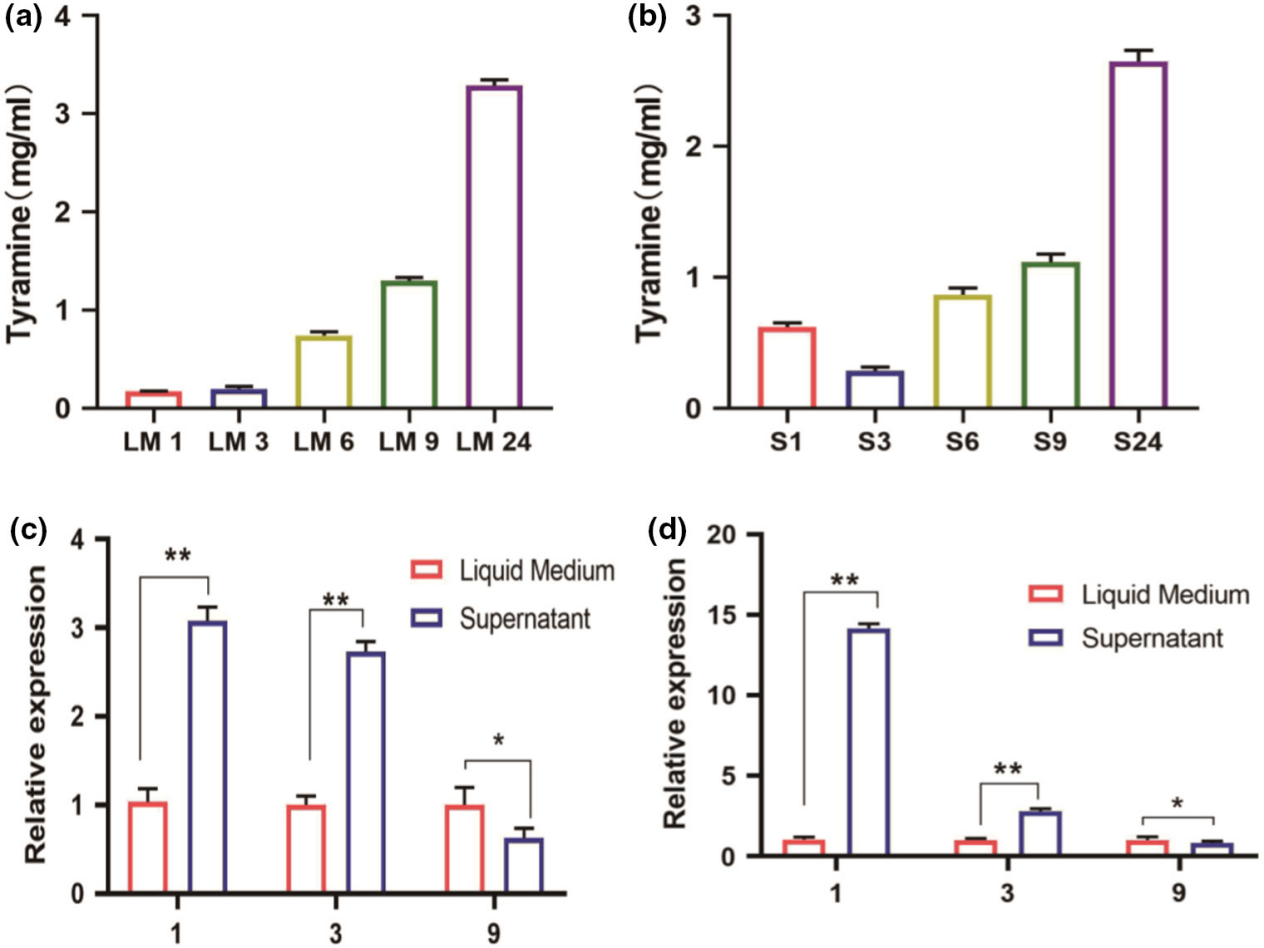
(a) *Enterococcus faecium* was inserted into the liquid medium to cultivate and detect tyramine content. “LM1, LM3, LM6, LM9 and LM24” in figure indicate *Enterococcus faecium* was inserted into the liquid medium to cultivate for 1, 3, 6, 9 and 24 h. (b) *Enterococcus faecium* was inserted into the sterile supernatant of LM1, LM3, LM6, LM9, and LM24 to cultivate detect tyramine content. “S1, S3, S6, S9 and S24” in figure indicate *Enterococcus faecium* was inserted into the sterile supernatant of LM1, LM3, LM6, LM9, and LM24 to cultivate for 1 h. (c) Expression of tyrDC genes in LM1 and S1, LM3 and S3, LM9 and S9. (d) Expression of tyrP genes in LM1 and S1, LM3 and S3, LM9 and S9. “*” in the figures indicate different groups with significant differences (*p* < .05). “**” in the figures indicate different groups with great significant differences (*p* < .01)

*Enterococcus faecium* is a tyramine‐producing bacterium in Sanchun ham, and quorum sensing can regulate the physiological activities of microorganisms through signal molecules. Whether the tyramine production process of *E. faecium* that produces tyrosine decarboxylase and decarboxylate with tyrosine, is regulated by quorum sensing signal molecules is an experiment aimed at this problem. Set up liquid medium groups (LM) and supernatant groups (S). LM was *E. faecium* inserted into the liquid medium to cultivate 1, 3, 6, 9 and 24 h. S was *E. faecium* inserted into the sterile supernatant of LM1, LM3, LM6, LM9, and LM24 to cultivate for 1 h. The tyramine content and gene expression level were comparative analyses in LM1 and S1, LM3 and S3, LM6 and S6, LM9 and S9, LM24 and S24. Figure 5a,b showed that the tyramine content in the LM1, LM3, and LM6 was significantly higher than S1, S3, and S6. The tyramine synthesis was inhibited in S9 and S24 compared with LM9 and LM24. Figure 5c,d showed that the levels of tyrDC and tyrP expression were enhanced in S1 and S3 compared with LM1 and LM3. The expression of tyrDC and tyrP in S9 was reduced compared with LM9. This result indicated the presence of signal molecules in the culture medium, which mediated tyramine production by *E. faecium*. The analysis of the results of Figures 2, 3, 4, 5 showed that tyrosine induced tyramine synthesis by *E. faecium*, which only occurred when the flora reached a certain level. We speculated that *E. faecium* synthesized microbial quorum sensing signal molecules in the course of tyramine synthesis. Therefore, nontargeted metabolomics were used to analyze the differential metabolites produced by *E. faecium*.

### 3.6 Nontargeted metabolomics analysis of differential metabolites produced by *E. faecium*

**FIGURE 6:**
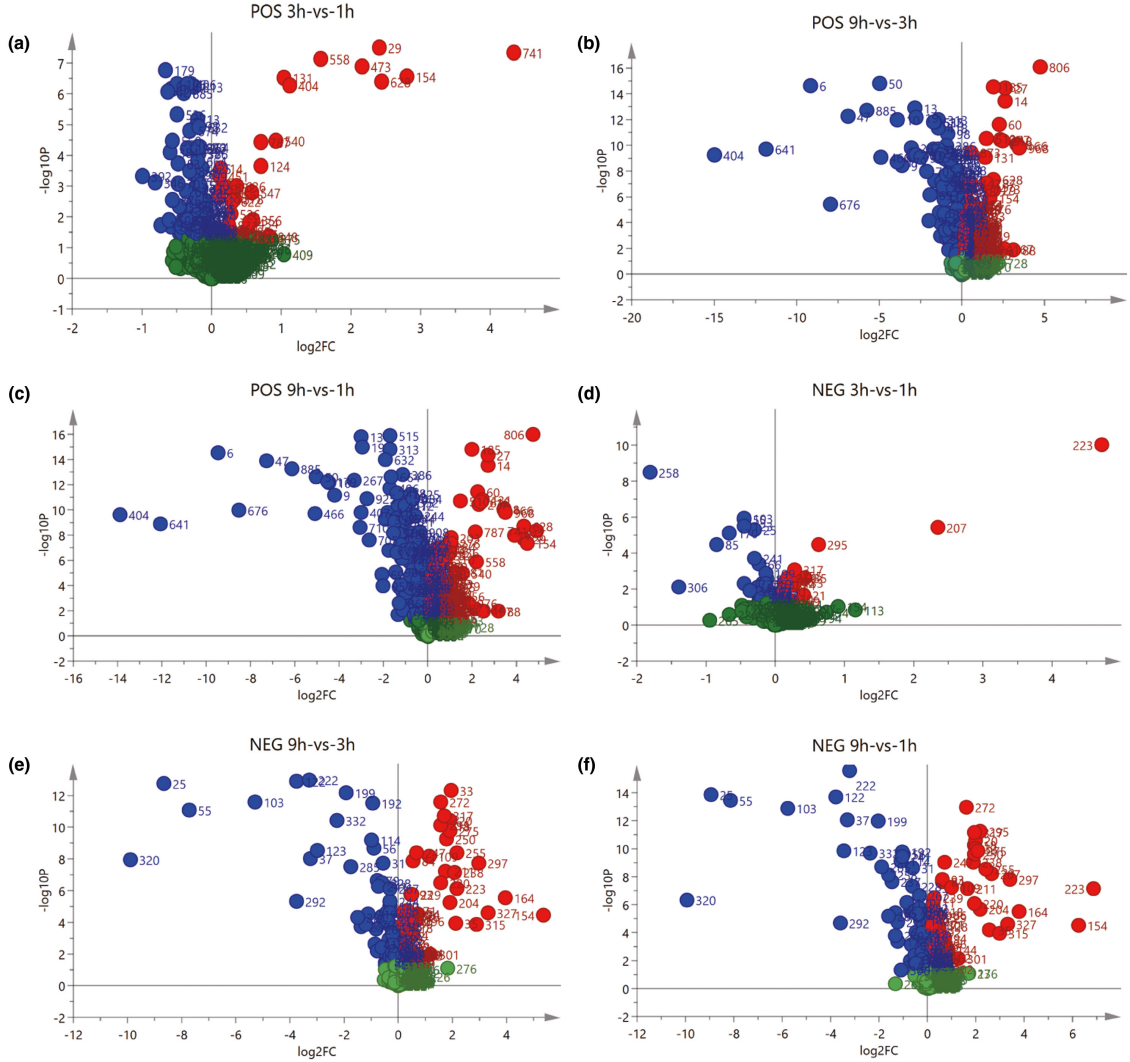
Volcano plot of metabolites of *Enterococcus faecium* under positive (a–c) and negative (d–f) ion mode. Green indicates *p* > .05. Red indicates *p* < .05 and fold change>1. Blue indicates *p* < .05 and fold change<1

**FIGURE 7:**
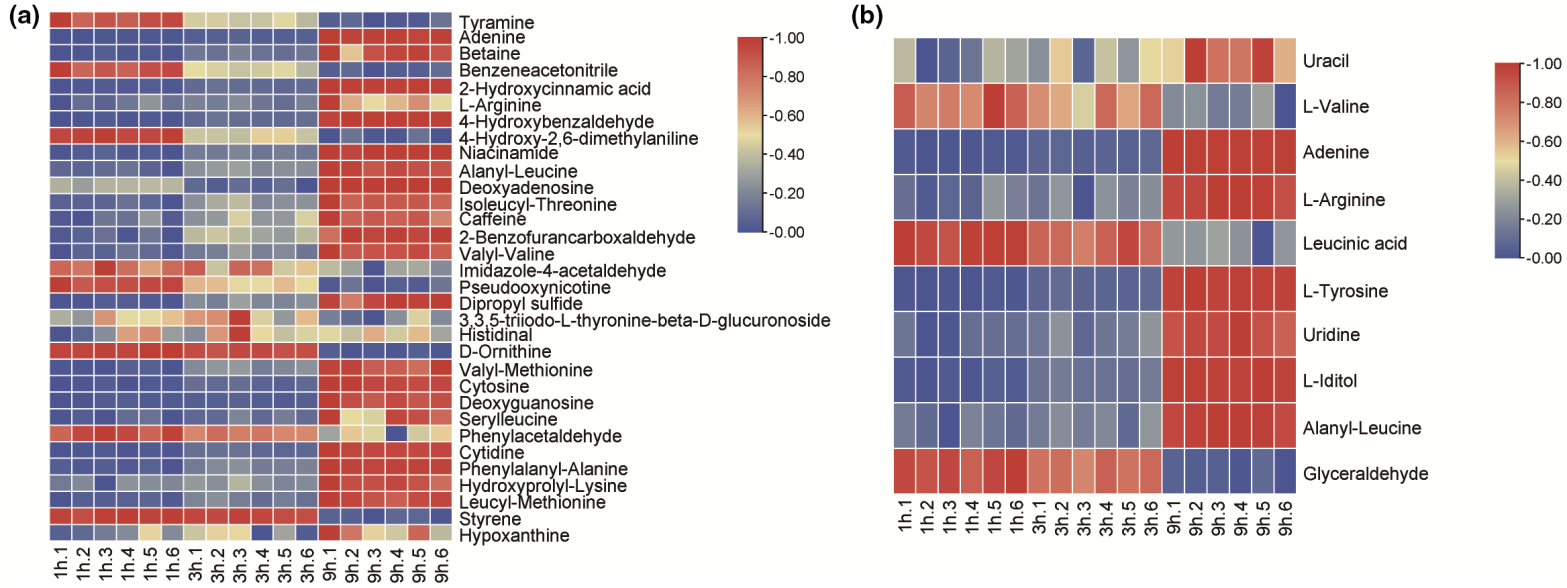
Heatmap of differential expressed metabolites of *Enterococcus faecium* in positive (a) and negative (b) ion modes

**FIGURE 8:**
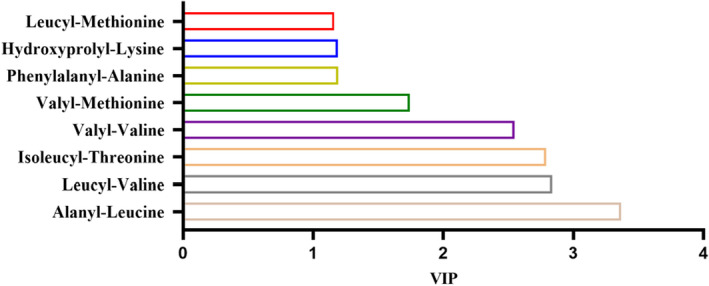
Differential peptide metabolites of *Enterococcus faecium* in positive ion mode

Figure 6 showed the volcano plot of metabolites in positive and negative ion mode in the supernatant of *E. faecium* cultured for different durations. Figure 7 showed the heatmap of differential metabolites in positive and negative ion mode with variable importance for the projection (VIP) higher than 1 in the supernatant. Figure 8 showed differential peptide metabolites in the supernatant of *E. faecium*. Differential metabolites mainly included amino acids and their derivatives, peptides, purines, nucleotides, and their derivatives. Tyramine was the main differential metabolite and the peptides were mainly dipeptides. Alanyl‐leucine was the peptide differential metabolite with the highest VIP value which is 3.367. Leucyl‐valine was the peptide differential metabolite with the second highest VIP value which is 2.837. The differential metabolites obtained using the same method in negative ion mode mainly included amino acids, alkaloids, nucleotides, and their derivatives, and tyrosine was the major differential amino acid metabolite. Alanyl‐leucine was the only peptide metabolite obtained in negative ion mode. Reports show that the quorum sensing signal molecules of Gram‐positive bacteria are mainly oligopeptides (Aggarwal & Federle, 2014). Therefore, we believe that alanyl‐leucine might be a quorum sensing signal molecule for tyramine synthesis by *E. faecium*.

### 3.7 Screening of signal molecule peptides

**FIGURE 9:**
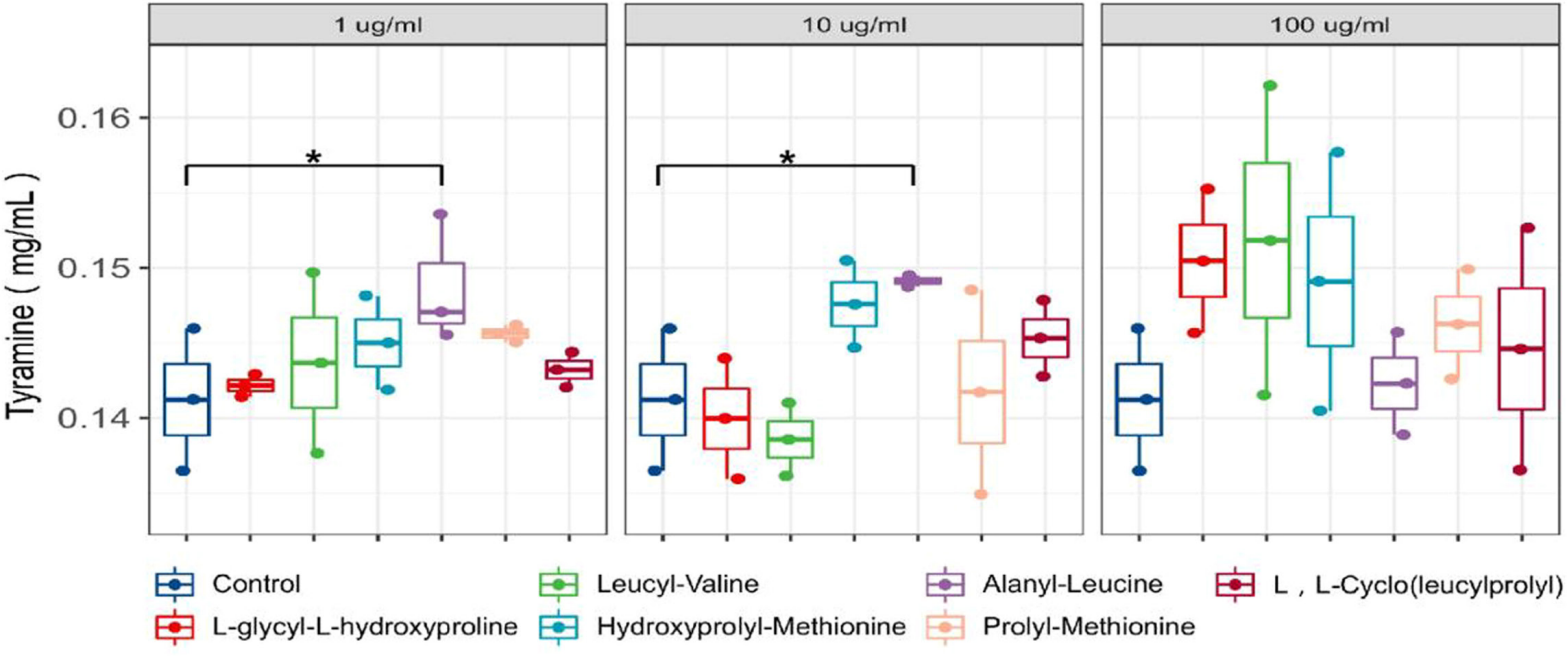
Effects of different synthetic peptides on tyramine production in *Enterococcus faecium*. “*” in the figures indicate different groups with significant differences (*p* < .05)

To identify peptide quorum sensing signaling molecules associated with tyramine production in *E. faecium*, six kinds of peptides were added to the liquid medium and the bacteria were cultured. Alanyl‐leucine and leucyl‐valine were the peptide differential metabolite in *E. faecium* culture supernatant. L‐glycyl‐L‐hydroxyproline, Hydroxyprolyl‐methionine, and Prolyl‐methionine were the peptide metabolite in *E. faecium* culture supernatant but not differential metabolite. Studies have shown that cyclodipeptide is a quorum sensing signal molecule, so the metabolites L, L‐cyclo (leucylprolyl) of *E*. *faecium* were selected for verification. The liquid medium without peptides was used as the blank control group for culture, and the tyramine content in the bacterial liquid was detected after 1 h culture. Figure 9 showed that the peptide concentration of 100 µg/ml was not significantly different from that of the control group (*p* > .05). The tyramine content was 0.156 ± 0.007 mg/ml in the control group and 0.169 ± 0.002 mg/ml in the alanyl‐leucine group when the peptide concentration was 10 µg/ml. The difference between the alanyl‐leucine group and the control group was significant (*p* < .05), there was no significant difference between the other groups and the control group (*p* > .05). The tyramine content of alanyl‐leucine group was significantly different from that of the control group (*p* < .05). The results showed that alanyl‐leucine was a quorum sensing signal peptide associated with tyramine production.

### 3.8 Effect of synthetic alanyl‐leucine on biogenic amine production by *E. faecium*

**FIGURE 10:**
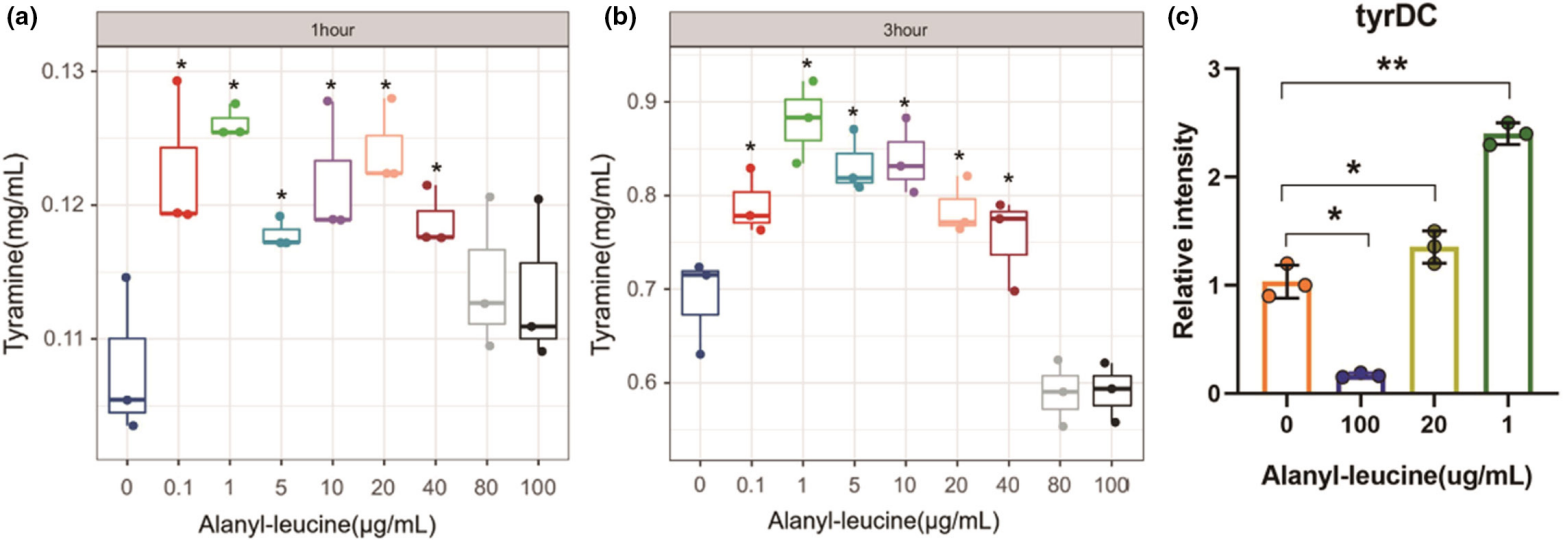
Effect of alanyl‐leucine on tyramine production in *Enterococcus faecium* cultivating with 1 h (a) and 3 h (b). Expression level of tyrDC gene of *Enterococcus faecium* supplemented with alanyl‐leucine for 3 h (c). “*” in the figures indicate different groups with significant differences (*p* < .05). “NS” in the figures indicates different groups with no significant differences (*p* > .05)

To further confirm the relationship between alanyl‐leucine and tyramine synthesis by *E*. *faecium*, we add alanyl‐leucine into the culture to evaluate the production of tyramine. Figure 10a showed that tyramine content did not differ significantly between the groups treated with 80 and 100 µg/ml alanyl‐leucine and the group not treated with tyramine after 1 h of culture (*p* > .05); the tyramine content of the group treated with 40 µg/ml alanyl‐leucine was significantly higher than that of the untreated group (*p* < .05). As shown in Figure 10b, tyramine content was significantly lower (*p* < .05) in groups treated with 80 and 100 µg/ml alanyl‐leucine for 3 h than in the group without alanyl‐leucine, although the difference between the groups treated with 40 and 0.0 µg/ml alanyl‐leucine was not significant. Compared to that in the group without alanyl‐leucine addition, tyramine content increased significantly with the addition of 20 µg/ml alanyl‐leucine. The above results indicated that a high concentration of alanyl‐leucine inhibited the synthesis of tyramine by *E. faecium*; when the concentration decreased to the threshold value, alanyl‐leucine promoted the production of tyramine by *E. faecium*. Figure 10c showed that compared to that in the control group, *tyrDC* was downregulated in the 100 µg/ml treatment group and upregulated in the 20 and 1 µg/ml treatment groups after 3 h of culture, suggesting that alanyl‐leucine regulated the expression of tyrosine decarboxylase (tyrDC) of *E. faecium*, and that high‐concentration alanyl‐leucine inhibited *tyrDC* expression, while low concentration close to the threshold promoted *tyrDC* expression. Thus, a low concentration of alanyl‐leucine (40 µg/ml) significantly stimulated the expression of tyrosine decarboxylase of *E. faecium*, which converted tyrosine into tyramine.

## 4 DISCUSSION

In this study, we isolated and identified a strain with high tyramine production from Sanchuan ham, and found a small peptide in this strain that can act as a QS molecule to regulate tyramine production. After synthesizing and adding the small peptide, tyramine production could be regulated effectively. The results suggest that small peptides, as QS molecules, play an important role in the regulation of biogenic amines. We showed that alanyl‐leucine was quorum sensing signal molecules of *E. faecium*, which can stimulate *tyrDC* and *tyrP* at very low concentration (40 µg/ml), promoting the conversion of tyrosine to tyramine by *E. faecium*. The strain used in this study was a tyramine‐producing strain isolated and purified from Sanchuan ham fermented for 1 year and was identified as *E*. *faecium*. Marcobal and others have reported that *E. faecium* RM58 synthesizes not only tyramine but also phenylethylamine (Marcobal et al., 2006). The *E. faecium* used in this study only produced tyramine, which might be because of differences in strains.

The presence of multiple biogenic amines in fermented ham has been confirmed. Liao used the HPLC method to detect the biogenic amines in Xuanwei Ham, tested phenethylamine, putrescine, cadaverine, tyramine, spermine, and spermidine, including spermine content (4.28 mg/100 g, which is the highest), and the content of total biogenic amine was 11.54 mg/100 g (Guozhou et al., 2011). Wang detected seven biological amines that were tryptamine, phenylethylamine, putrescine, tyramine, cadaverine, spermine, and spermidine in Xuanwei ham samples and the content of spermine was the highest at each processing stage, followed by tyramine and spermidine (Guiying et al., 2012). Phenylethylamine and putrescine contents increased significantly (*p* < .05), reached the highest value at the middle maturity (250 days), and then decreased (*p* > .05). The total amount of biogenic amines increased with the processing and reached the maximum value of 11.51 mg/100 g at the middle maturity stage (250 days); then, the total amount of biogenic amines tended to decrease, but the difference was not significant (*p* > .05). These results showed that seven kinds of biological amines were detected in Rugao ham, including tryptamine, putrescine, phenylethylamine, cadaverine, tyramine, spermine, and spermidine, among which spermine content was the highest and the total biological amines reached the maximum value of 14.97 mg/100 g at 55 days of drying and then decreased continuously for 325 days to 2.07 mg/100 g (Wei et al., 2009). The main accumulation period of biogenic amines was in the heap‐covering fermentation period, and the mass concentration of total biogenic amines was the highest in the later fermentation period. Italian dry‐cured hams were found to have the highest levels of tyramine, followed by spermine (Virgili et al., 2007). The content of biogenic amines in Italian smoked ham was determined and the total biogenic amines content reached 79.02 mg/kg (Martuscelli et al., 2009). The highest content of total biogenic amines was 140.6 mg/kg in Portuguese ham cured for 10 months (Alfaia et al., 2004). Joong‐Seok Min quantitatively analyzed the biogenic amines in processed and unprocessed foods from animal sources in the Korean domestic market and detected histamine, tryptamine, putrescine, tyramine, spermine, and spermidine in ham (Min et al., 2004). These results all indicate that there are harmful biogenic amines in ham, and these amines will gradually accumulate in human body with the increase in consumption and eventually affect human health. Therefore, it is necessary to monitor the content of biogenic amines in commercial ham. The type and content of biogenic amines were changed by associated with raw materials and processing technology in ham. And different manufacturing processes also generated changes of biogenic amines in the same ham. All the variation was associated with the dominant microorganism during the ham fermentation process. With the change in different processing technology and processing environment, the dominant microorganisms in ham also changed. Some microorganisms had the corresponding amino acid decarboxylase gene, which could decarboxylate with the corresponding amino acid to produce biogenic amines. The production of biogenic amines by these microorganisms may also be related to the different expression and regulation of genes caused by the transduction of some signal molecules between microorganisms.

Biogenic amine production is often observed in the process of ham fermentation, such as Xuanwei ham, Spanish ham, and Rugao ham, but the detection of biogenic amine in Sanchuan ham is less studied. In this study, the accumulation of biogenic amines in eight kinds of commercial ham was detected, especially in Sanchuan ham. It was found that Sanchuan ham had a high accumulation of biogenic amines, which provided a material basis for the research on the regulation of biogenic amines production. A strain with high tyramine production was obtained through isolation and identification. Compared with other hams, it was found that this strain was the main amine‐producing bacterium of ham. The results of the fermentation environment study were similar to those reported in another literature, indicating that the strain played a very important role in the production of biogenic amines. It has been reported that the QS molecule of positive bacteria is mainly AIP (Wu et al., 2020). Similar to other reports, this study found a small peptide as a QS molecule, which was discovered for the first time in Sanchuan ham. This study proved that the small peptide is a potential QS molecule and can regulate the production of biological amines by various means. Figures 1 and 2 show that tyramine accumulated rapidly in the logarithmic growth phase of *E. faecium*, which is following the growth characteristics of the strain. High temperature promoted the production of tyramine by *E. faecium*, while low temperature showed the opposite effect. The acidic environment promoted the production of tyramine by *E. faecium*, whereas a high concentration of NaCl significantly inhibited its production. Microbial growth, biochemical reaction, metabolite production, and the production rate of biogenic amine also increased with temperature. In contrast, at low temperatures, poor microbial growth and reduced enzyme activity minimized the accumulation of biogenic amine (Prester et al., 2009). pH is an important factor that affects the activity of amino acid decarboxylase via two mechanisms. First, pH influences the growth of microorganisms; second, it affects the production and activity of enzymes. Bacteria are more likely to be stimulated to produce decarboxylase at low pH, which is a part of the bacterial defense mechanism against acidity (Perez et al., 2015). In addition, although low pH promoted the production of tyramine by *E. faecium* in this study, its production was maximum when the pH for tyrosine decarboxylase activity was optimum, indicating that tyramine production was determined by the activity of tyrosine decarboxylase. Salt is used to prevent the growth of microorganisms and control the spoilage of ham. Sodium chloride affects the activity of amino acid decarboxylase and hence biogenic amine production. Suzzi and Gardini showed that the accumulation of biogenic amines in fermented sausages decreased significantly with an increase in NaCl concentration (Suzzi & Gardini, 2003). Mutz and others showed that in higher NaCl clusters, the salt concentration had an inhibitory property in biogenic amines formation (Mutz et al., 2021). High salt content inhibited the growth of amine‐producing bacteria and the activity of amino acid decarboxylase (Loizzo et al., 2013).

Figure 3 shows that tyrosine was the substrate used for tyramine production by *E. faecium*, and that the production depended on tyrosine concentration. The tyramine content increased with tyrosine concentration. Various biochemical and physical changes occur during the fermentation and maturation of ham. The enzymes produced from muscle tissues and microorganisms hydrolyze proteins, producing small peptides and free amino acids, which are precursors of biogenic amines produced by microorganisms with amino acid decarboxylase activity (Freiding et al., 2011; Latorre‐Moratalla et al., 2014). External environment factors stimulate *E. faecium* to produce tyramine from tyrosine. This process is completed inside the cell, where the substrate tyrosine is transported into the cell by the reverse transport of cell membrane. Tyrosine is then decarboxylated into tyramine by the tyrosine decarboxylase secreted by the cell, which is transported outside the cell by the transporter. Tyrosine decarboxylases and tyrosine transporter play a major role in this process. Tyrosine is absorbed from the culture medium by tyrosine transporter and then decarboxylated by tyrosine decarboxylases to produce tyramine (Wolken et al., 2006). TyrP is released into the cells after being bound to tyramine. The physiological function of this pathway is to generate proton power at the expense of free energy released by decarboxylation (Huang et al., 2022). Membrane potential and pH gradient generated in the pathway are two components of proton dynamics. The conversion of tyrP changes the membrane potential, as monovalent and positively charged tyramine is exchanged for uncharged tyrosine. TyrDC‐catalyzed tyrosine decarboxylation results in the consumption of a proton, which alkalizes the cytoplasm relative to the external medium (Bargossi et al., 2017). When an external tyrosine molecule is converted into an external tyramine molecule every time, a positive charge is transferred to the cell membrane and a proton is removed from the cytoplasm, which is equivalent to pumping of a proton through the cell membrane. Therefore, this pathway is an indirect proton pump. In addition, this pathway may play a role in maintaining cytoplasmic pH homeostasis and acid stress resistance via alkalization postdecarboxylation (Sobczak & Lolkema, 2005).

Bacteria can spontaneously produce and release specific signal molecules and can sense changes in their concentration to regulate group behavior (Jimenez et al., 2012). This regulation system is called quorum sensing. The substance that bacteria use to communicate with each other in the quorum sensing system is called the signal molecule (Boo et al., 2021). The N‐acetylhomoserine lactone (AHLS), mediated by the LuxI/LuxR system, is commonly used as a signal molecule by Gram‐negative bacteria, while Gram‐positive bacteria use autoinducing peptides (AIP) for quorum sensing (Camilli, 2006; Machado et al., 2019). AIP secretion increased with cell density; the signal cannot freely diffuse across the cell membrane, it is usually transported out of the cell via an AIP‐binding cassette (ABC) transporter (Verbeke et al., 2021). AIP needs relaying by two‐component histidine kinase; signal transduction resulting from cascade phosphorylation ultimately activates DNA‐binding proteins that influence the transcription of specific genes (Frederick et al., 2017). For the presence and quantitative analysis of quorum sensing peptides (QSPs), organic solvent precipitation, ultrafiltration, and solid‐phase extraction are used to prepare samples extraction; ultraviolet detection and mass spectrometry combined with the high‐performance liquid chromatography system are the main detection methods (Debunne et al., 2018). According to the Quorumpeps database, 10 QSPs are currently known to be produced by *Enterococcus faecalis* (Evelien et al., 2013). Frederick and others used UHPLC‐MS detection and quantification of nine QSPs of *E*. *faecalis* RNPP‐type quorum sensing peptides in bacterial culture media (Frederick et al., 2018). In this study, we found that alanyl‐leucine, which is a small peptide, could act as a signal molecule in the culture medium of *E. faecium* that regulates the production of biogenic amines. Previous study reported that dipeptide could regulate microbial growth (Xu et al., 2021). In this study, we also found that dipeptide alanyl‐leucine could regulate *E*. *faecium* growth. But no study has report that dipeptide could be a quorum sensing molecule and we were the first time to provide a date to verify in this study. The cell‐signaling peptides could regulation of competence, sporulation, and biofilm formation (Anju et al., 2018). The alanyl‐leucine can be regarded as a cell‐signaling peptides maybe have a similar function. And further studies are also required to verify the function of the small peptides in bacterial.

## 5 CONCLUSION

In this study, a high‐yielding tyramine strain of Sanchuan ham was isolated and identified, which is *E. faecium*. Culture conditions considerably affect the synthesis of tyramine by *E. faecium*. Tyrosine can induce the production of tyramine by *E. faecium* in a concentration‐dependent manner. The differential peptide metabolites of *E. faecium*, which is alanyl‐leucine, were analyzed using nontargeted metabolomics. Experiments using artificially synthesized alanyl‐leucine have shown that alanyl‐leucine is a quorum sensing signal molecule, which can regulate the synthesis of tyramine by *E. faecium* at extremely low concentration (≤40 µg/ml). Based on the analysis of metabolomics, the quorum sensing signal molecules related to tyramine production of *E. faecium* were obtained, which will be beneficial to control tyramine production of *E. faecium* by inhibiting quorum sensing in the future, thereby inhibiting the content of tyramine in ham, and providing a new idea for controlling the quality of ham.

## CONFLICT OF INTEREST

The authors declare that the research was conducted in the absence of any commercial or financial relationships that could be construed as a potential conflict of interest.

## Data Availability

The data that support the findings of this study are available from the corresponding author upon reasonable request.

## References

[fsn32820-bib-0001] Aggarwal, C. , & Federle, M. J. (2014). Peptide pheromones and their protein receptors: Cellular signaling in gram‐positive bacteria. Molecular Life Sciences, 1–14. 10.1007/978-1-4614-6436-5

[fsn32820-bib-0002] Alfaia, C. M. , Castro, M. F. , Reis, V. A. , Prates, J. M. , de Almeida, I. T. , Correia, A. D. , & Dias, M. A. (2004). Changes in the profile of free amino acids and biogenic amines during the extended short ripening of Portuguese dry‐cured ham. Food Science and Technology International, 10(5), 297–304. 10.1177/1082013204047597

[fsn32820-bib-0003] Alvatez, M. A. , & Moreno‐Arribas, M. V. (2014). The problem of biogenic amines in fermented foods and the use of potential biogenic amine‐degrading microorganisms as a solution. Trends in Food Science and Technology, 39(2), 146–155.

[fsn32820-bib-0004] Anju, S. , Aparna, Y. , Bhima, B. , & Sarada, J. (2018). Novel insights on the bacillus quorum sensing mechanism: Its role in competence, virulence, sporulation and biofilm formation. In B. Pallaval Veera (Ed.), Implication of quorum sensing system in biofilm formation and virulence (pp. 313–327). Springer Singapore.

[fsn32820-bib-0005] Ashaolu, T. J. , Khalifa, I. , Mesak, M. A. , Lorenzo, J. M. , & Farag, M. A. (2021). A comprehensive review of the role of microorganisms on texture change, flavor and biogenic amines formation in fermented meat with their action mechanisms and safety. Critical Reviews in Food Science and Nutrition, 1–18. 10.1080/10408398.2021.1929059 34014126

[fsn32820-bib-0006] Bargossi, E. , Tabanelli, G. , Montanari, C. , Gatto, V. , Chinnici, F. , Gardini, F. , & Torriani, S. (2017). Growth, biogenic amine production and tyrDC transcription of Enterococcus faecalis in synthetic medium containing defined amino acid concentrations. Journal of Applied Microbiology, 122(4), 1078–1091. 28117533 10.1111/jam.13406

[fsn32820-bib-0007] Biji, K. B. , Ravishankar, C. N. , Venkateswarlu, R. , Mohan, C. O. , & Gopal, T. K. S. (2016). Biogenic amines in seafood: A review. Journal of Food Science and Technology, 53(5), 2210–2218. 10.1007/s13197-016-2224-x 27407186 PMC4921096

[fsn32820-bib-0008] Boo, A. , Ledesma Amaro, R. , & Stan, G.‐B. (2021). Quorum sensing in synthetic biology: A review. Current Opinion in Systems Biology, 28, 100378. 10.1016/j.coisb.2021.100378

[fsn32820-bib-0009] Brink, B. T. , Damink, C. T. , Joosten, H. M. L. J. , & Huis in 't Veld, J. H. J. (1990). Occurrence and formation of biologically active amines in foods. International Journal of Food Microbiology, 11(1), 73–84. 10.1016/0168-1605(90)90040-C 2223522

[fsn32820-bib-0010] Camilli, A. (2006). Bacterial small‐molecule signaling pathways. Science, 311(5764), 1113–1116. 16497924 10.1126/science.1121357 PMC2776824

[fsn32820-bib-0011] Debunne, N. , Verbeke, F. , Janssens, Y. , Wynendaele, E. , & Spiegeleer, B. D. (2018). Chromatography of quorum sensing peptides: An important functional class of the bacterial peptidome. Chromatographia, 8, 25–40. 10.1007/s10337-017-3411-2

[fsn32820-bib-0012] Evelien, W. , Antoon, B. , Joachim, N. , Matthias, D. H. , Sofie, S. , Nathalie, B. , & Bart, D. S. (2013). Quorumpeps database: Chemical space, microbial origin and functionality of quorum sensing peptides. Nucleic Acids Research, 41(D1), 655–659. 10.1093/nar/gks1137 PMC3531179 23180797

[fsn32820-bib-0013] Fan, Q. , Wang, H. , Mao, C. , Li, J. , Zhang, X. , Grenier, D. , Yi, L. I. , & Wang, Y. (2022). Structure and signal regulation mechanism of interspecies and interkingdom quorum sensing system receptors. Journal of Agricultural Food Chemistry, 70, 429–445. 10.1021/acs.jafc.1c04751 34989570

[fsn32820-bib-0014] Frederick, V. , Nathan, D. , Yorick, J. , Liesa, T. , Evelien, W. , Petra, R. , & Bart, D. S. (2018). Detection and quantification of *Enterococcus faecalis* RNPP‐type quorum sensing peptides in bacterial culture media by UHPLC‐MS. Journal of Pharmaceutical Biomedical Analysis, 160, 55–63. 30059814 10.1016/j.jpba.2018.07.019

[fsn32820-bib-0015] Frederick, V. , Severine, D. C. , Nathan, D. , Yorick, J. , Evelien, W. , Christophe, V. D. W. , & Bart, D. S. (2017). Peptides as quorum sensing molecules: Measurement techniques and obtained levels in vitro and in vivo. Frontiers in Neuroscience, 11, 183. 28446863 10.3389/fnins.2017.00183 PMC5388746

[fsn32820-bib-0016] Freiding, S. , Gutsche, K. A. , Ehrmann, M. A. , & Vogel, R. F. (2011). Genetic screening of Lactobacillus sakei and Lactobacillus curvatus strains for their peptidolytic system and amino acid metabolism, and comparison of their volatilomes in a model system. Systematic and Applied Microbiology, 34(5), 311–320. 10.1016/j.syapm.2010.12.006 21570226

[fsn32820-bib-0017] Fu, L. , Wang, C. , Liu, N. , Ma, A. , & Wang, Y. (2018). Quorum sensing system‐regulated genes affect the spoilage potential of Shewanella baltica. Food Research International, 107, 1–9. 10.1016/j.foodres.2018.01.067 29580465

[fsn32820-bib-0018] Guiying, W. , Hong, Y. , Jinxuan, C. , Zongfang, S. , & Guozhou, L. (2012). Changes of biogenic amines during processing of Xuanwei Ham. Food and Fermentation Industries, 38(004), 192–195.

[fsn32820-bib-0019] Guozhou, L. , Guiying, W. , Jinxuan, C. , & Zhibin, C. (2011). Detection of biogenic amines in Xuanwei Ham by HPLC. Food and Fermentation Industries, 37(012), 130–132.

[fsn32820-bib-0020] Halasz, A. , Barath, A. , Simon‐Sarkadi, L. , & Holzapfel, W. (1994). Biogenic amines and their production by microorganisms in food. Trends in Food Science and Technology, 5(2), 42–49. 10.1016/0924-2244(94)90070-1

[fsn32820-bib-0021] Hu, Y. , Xue, Q. , Li, Z. , Zhang, Y. , & Li, S. (2018). The change rule of biogenic amines during the processing of Sanchuan Ham. Journal of Light Industry, 033(005), 1–8.

[fsn32820-bib-0022] Huang, Y. , Yu, H. , Lu, S. , Zou, L. , Tang, Z. , Zeng, T. , & Tang, J. (2022). Effect and mechanism of ferulic acid inclusion complexes on tyramine production by Enterobacter hormaechei MW386398 in smoked horsemeat sausages. Food Bioscience, 46, 101520. 10.1016/j.fbio.2021.101520

[fsn32820-bib-0023] Huichao, Z. , Binbin, L. , Lili, Z. , Qingling, W. , Baokun, L. , & Shiling, L. (2019). The effects of amine oxidase‐producing starter culture on biogenic amine accumulation in traditional Chinese smoked horsemeat sausages. Journal of Food Safety, 39(3), 1–7. 10.1111/jfs.12638

[fsn32820-bib-0024] Jaguey‐Hernández, Y. , Aguilar‐Arteaga, K. , Ojeda‐Ramirez, D. , Añorve‐Morga, J. , González‐Olivares, L. G. , & Castañeda‐Ovando, A. (2021). Biogenic amines levels in food processing: Efforts for their control in foodstuffs. Food Research International, 144(9), 110341. 10.1016/j.foodres.2021.110341 34053537

[fsn32820-bib-0025] Jimenez, P. N. , Koch, G. , Thompson, J. A. , Xavier, K. B. , Cool, R. H. , & Quax, W. J. (2012). The multiple signaling systems regulating virulence in Pseudomonas aeruginosa. Microbiology and Molecular Biology Reviews, 76(1), 46–65. 10.1128/MMBR.05007-11 22390972 PMC3294424

[fsn32820-bib-0026] Kareb, O. , & Ader, M. (2020). Quorum sensing circuits in the communicating mechanisms of bacteria and its implication in the biosynthesis of bacteriocins by lactic acid bacteria: A review. Probiotics Antimicrobial Proteins, 12(1), 5–17. 10.1007/s12602-019-09555-4 31104210

[fsn32820-bib-0027] Latorre‐Moratalla, M. L. , Bover‐Cid, S. , Bosch‐Fusté, J. , Veciana‐Nogués, M. T. , & Vidal‐Carou, M. C. (2014). Amino acid availability as an influential factor on the biogenic amine formation in dry fermented sausages. Food Control, 36(1), 76–81. 10.1016/j.foodcont.2013.07.038

[fsn32820-bib-0028] Lin, F. , Cai, F. , Luo, B. , Gu, R. , Ahmed, S. , & Long, C. (2020). Variation of microbiological and biochemical profiles of laowo dry‐cured ham, an indigenous fermented food, during ripening by GC‐TOF‐MS and UPLC‐QTOF‐MS. Journal of Agricultural and Food Chemistry, 63(33), 8925–8935. 10.1021/acs.jafc.0c03254 32706588

[fsn32820-bib-0029] Liu, S. , Wang, G. , Xiao, Z. , Pu, Y. , Ge, C. , & Liao, G. (2019). ^1^H‐NMR‐based water‐soluble low molecular weight compound characterization and free fatty acid composition of five kinds of Yunnan dry‐cured hams. LWT‐Food Science and Technology, 108, 174–182. 10.1016/j.lwt.2019.03.043

[fsn32820-bib-0030] Loizzo, M. R. , Menichini, F. , Picci, N. , Puoci, F. , Spizzirri, U. G. , & Restuccia, D. (2013). Technological aspects and analytical determination of biogenic amines in cheese. Trends in Food Science and Technology, 30(1), 38–55. 10.1016/j.tifs.2012.11.005

[fsn32820-bib-0031] Lu, S. L. , Jiang, C. H. , Xu, X. L. , Xu, C. J. , Li, K. X. , & Shu, R. H. (2015). Improved screening procedure for biogenic amine production by lactic acid bacteria and Enterobacteria. Czech Journal of Food Sciences, 33(1), 19–26. 10.17221/197/2014-CJFS 10598112

[fsn32820-bib-0032] Machado, I. , Silva, L. R. , Giaouris, E. D. , Melo, L. F. , & Simes, M. (2019). Quorum sensing in food spoilage and natural‐based strategies for its inhibition. Food Research International, 127, 1–36. 10.1016/j.foodres.2019.108754 31882100

[fsn32820-bib-0033] Marcobal, A. , Rivas, B. D. L. , & Muoz, R. (2006). First genetic characterization of a bacterial β‐phenylethylamine biosynthetic enzyme in Enterococcus faecium RM58. FEMS Microbiology Letters, 258(1), 1–19. 16630269 10.1111/j.1574-6968.2006.00206.x

[fsn32820-bib-0034] Martínez‐Onandi, N. , Sánchez, C. , Nuñez, M. , & Picon, A. (2019). Microbiota of Iberian dry‐cured ham as influenced by chemical composition, high pressure processing and prolonged refrigerated storage. Food Microbiology, 80, 62–69. 10.1016/j.fm.2019.01.002 30704597

[fsn32820-bib-0035] Martuscelli, M. , Pittia, P. , Casamassima, L. M. , Marietta, A. C. , Lupieri, L. , & Neri, L. (2009). Effect of intensity of smoking treatment on the free amino acids and biogenic amines occurrence in dry cured ham. Food Chemistry, 116(4), 955–962. 10.1016/j.foodchem.2009.03.061

[fsn32820-bib-0036] Miller, M. B. , & Bassler, B. L. (2001). Quorum sensing in bacteria. Annual Review of Microbiology, 55, 165–199. 10.1146/annurev.micro.55.1.165 11544353

[fsn32820-bib-0037] Min, J.‐S. , Lee, S.‐O. , Jang, A. , Lee, M. , & Kim, Y. (2004). Quantitative analysis of biogenic amines in raw and processed foods of animal origin on Korean domestic market. Asian Australasian Journal of Animal Sciences, 17(12), 1764–1768. 10.5713/ajas.2004.1764

[fsn32820-bib-0038] Moret, S. , & Conte, L. S. (1996). High‐performance liquid chromatographic evaluation of biogenic amines in foods. An analysis of different methods of sample preparation in relation to food characteristics. Journal of Chromatography A, 729(1–2), 363–369. 9004961 10.1016/0021-9673(95)00961-2

[fsn32820-bib-0039] Mukherjee, S. , & Bassler, B. L. (2019). Bacterial quorum sensing in complex and dynamically changing environments. Nature Reviews Microbiology, 17, 371–382. 10.1038/s41579-019-0186-5 30944413 PMC6615036

[fsn32820-bib-0040] Mutz, Y. S. , Kaic Alves Rosario, D. , Alves de Aguiar Bernardo, Y. , Paulo Vieira, C. , Vilela Pinto Moreira, R. , Bernardes, P. C. , & Conte‐Junior, C. A. (2021). Unravelling the relation between natural microbiota and biogenic amines in Brazilian dry‐cured loin: A chemometric approach. International Journal of Food Science & Technology, 57, 1621–1629.

[fsn32820-bib-0041] Ozogul, F. , & Hamed, I. (2017). The importance of lactic acid bacteria for the prevention of bacterial growth and their biogenic amines formation: A review. Critical Reviews in Food Science and Nutrition, 58(10), 1–39. 10.1080/10408398.2016.1277972 28128651

[fsn32820-bib-0042] Perez, M. , Calles‐Enríquez, M. , Nes, I. , Martin, M. C. , Fernandez, M. , Ladero, V. , & Alvarez, M. A. (2015). Tyramine biosynthesis is transcriptionally induced at low pH and improves the fitness of Enterococcus faecalis in acidic environments. Applied Microbiology Biotechnology, 99(8), 3547–3558. 10.1007/s00253-014-6301-7 25529314

[fsn32820-bib-0043] Perin, L. M. , & Nero, L. A. (2017). The relevance of biogenic amines in dairy products. In Dairy in human health and disease across the lifespan (pp. 169–182). Academic Press.

[fsn32820-bib-0044] Prester, L. , Macan, J. , Varnai, V. M. , Orct, T. , Vukusic, J. , & Kipcic, D. (2009). Endotoxin and biogenic amine levels in Atlantic mackerel (Scomber scombrus), sardine (Sardina pilchardus) and Mediterranean hake (Merluccius merluccius) stored at 22 degrees C. Food Additives & Contaminants, 26(3), 355–362. 19680909 10.1080/02652030802520878

[fsn32820-bib-0045] Santos, M. H. S. (1996). Biogenic amines: Their importance in foods. International Journal of Food Microbiology, 29(2–3), 213–231. 10.1016/0168-1605(95)00032-1 8796424

[fsn32820-bib-0046] Sobczak, I. , & Lolkema, J. S. (2005). The 2‐Hydroxycarboxylate transporter family: Physiology, structure, and mechanism. Microbiology and Molecular Biology Reviews, 69(4), 665–695. 10.1128/MMBR.69.4.665-695.2005 16339740 PMC1306803

[fsn32820-bib-0047] Suzzi, G. , & Gardini, F. (2003). Biogenic amines in dry fermented sausages: A review. International Journal of Food Microbiology, 88(1), 41–54. 10.1016/S0168-1605(03)00080-1 14527784

[fsn32820-bib-0048] Vasconcelos, H. , Coelho, L. C. C. , Matias, A. , Saraiva, C. , Jorge, P. A. S. , & de Almeida, J. M. M. M. (2021). Biosensors for biogenic amines: A review. Biosensors, 11(3), 82. 10.3390/bios11030082 33805834 PMC8000219

[fsn32820-bib-0049] Verbeke, F. , Debunne, N. , Janssens, Y. , Spiegeleer, B. D. , & Wynendaele, E. (2021). A bioanalytical screening method for *Enterococcus faecalis* RNPP‐type quorum sensing peptides in murine feces. Bioanalysis, 14(3), 151–167. 10.4155/bio-2021-0225 35014887

[fsn32820-bib-0050] Virgili, R. , Saccani, G. , Gabba, L. , Tanzi, E. , & Bordini, C. S. (2007). Changes of free amino acids and biogenic amines during extended ageing of Italian dry‐cured ham. LWT‐Food Science and Technology, 40(5), 871–878. 10.1016/j.lwt.2006.03.024

[fsn32820-bib-0051] Wang, Y. , Liu, Y. , Huang, X. , Xiao, Z. , Yang, Y. , Yu, Q. , Chen, S. , He, L. I. , Liu, A. , Liu, S. , Zou, L. , & Yang, Y. (2021). A review on mechanistic overview on the formation of toxic substances during the traditional fermented food processing. Food Reviews International, 1, 1–18. 10.1080/87559129.2021.1933021

[fsn32820-bib-0052] Wei, F. , Xu, X. , Zhou, G. , Zhao, G. , Li, C. , Zhang, Y. , Chen, L. , & Qi, J. (2009). Irradiated Chinese Rugao ham: Changes in volatile N‐nitrosamine, biogenic amine and residual nitrite during ripening and post‐ripening. Meat Science, 81(3), 451–455. 10.1016/j.meatsci.2008.09.005 22064282

[fsn32820-bib-0053] Wolken, W. A. M. , Lucas, P. M. , Lonvaud‐Funel, A. , & Lolkema, J. S. (2006). The mechanism of the tyrosine transporter TyrP supports a proton motive tyrosine decarboxylation pathway in Lactobacillus brevis. Journal of Bacteriology, 188(6), 2198–2206. 16513749 10.1128/JB.188.6.2198-2206.2006 PMC1428153

[fsn32820-bib-0054] Wu, S. , Liu, J. , Liu, C. , Yang, A. , & Qiao, J. (2020). Quorum sensing for population‐level control of bacteria and potential therapeutic applications. Cellular and Molecular Life Sciences, 77(7), 1319–1343. 10.1007/s00018-019-03326-8 31612240 PMC11104945

[fsn32820-bib-0055] Xu, Q. , Hu, M. , Li, M. , Hou, J. , Zhang, X. , Gao, Y. A. , Chachar, B. , & Li, X. (2021). Dietary bioactive peptide alanyl‐glutamine attenuates dextran sodium sulfate‐induced colitis by modulating gut microbiota. Oxidative Medicine and Cellular Longevity, 2021, 5543003. 10.1155/2021/5543003 34046146 PMC8128544

[fsn32820-bib-0056] Zaman, M. Z. , Bakar, F. A. , Jinap, S. , & Bakar, J. (2011). Novel starter cultures to inhibit biogenic amines accumulation during fish sauce fermentation. International Journal of Food Microbiology, 145(1), 84–91. 10.1016/j.ijfoodmicro.2010.11.031 21183239

[fsn32820-bib-0057] Zezong, L. (2018). Effect of dominant microorganisms on biogenic amines in Sanchuan Ham. Yunnan Agricultural University.

[fsn32820-bib-0058] Zhu, J. , Zhao, A. , Feng, L. , & Gao, H. (2016). Quorum sensing signals affect spoilage of refrigerated large yellow croaker (Pseudosciaena crocea) by Shewanella baltica. International Journal of Food Microbiology, 217(18), 146–155. 10.1016/j.ijfoodmicro.2015.10.020 26519730

[fsn32820-bib-0059] Zhu, S. , Wu, H. , Zeng, M. , Liu, Z. , & Wang, Y. (2015). The involvement of bacterial quorum sensing in the spoilage of refrigerated Litopenaeus vannamei. International Journal of Food Microbiology, 192(2), 26–33. 10.1016/j.ijfoodmicro.2014.09.029 25305441

